# Handspinning Enabled Highly Concentrated Carbon Nanotubes with Controlled Orientation in Nanofibers

**DOI:** 10.1038/srep37590

**Published:** 2016-11-23

**Authors:** Hoik Lee, Kei Watanabe, Myungwoong Kim, Mayakrishnan Gopiraman, Kyung-Hun Song, Jung Soon Lee, Ick Soo Kim

**Affiliations:** 1 Nano Fusion Technology Research Lab, Division of Frontier Fibers, Institute for Fiber Engineering (IFES), Interdisciplinary Cluster for Cutting Edge Research (ICCER), Shinshu University 3-15-1, Tokida, Ueda, Nagono 386-8567, Japan; 2 Department of Chemistry, Inha University, Incheon 22212, Korea; 3 Department of Clothing and Textiles, Pai Chai University, Daejeon 35345, Korea; 4 Department of Clothing and Textiles, Chungnam National University, Daejeon 34134, Korea

## Abstract

The novel method, *handspinning* (HS), was invented by mimicking commonly observed methods in our daily lives. The use of HS allows us to fabricate carbon nanotube-reinforced nanofibers (CNT-reinforced nanofibers) by addressing three significant challenges: (i) the difficulty of forming nanofibers at high concentrations of CNTs, (ii) aggregation of the CNTs, and (iii) control of the orientation of the CNTs. The handspun nanofibers showed better physical properties than fibers fabricated by conventional methods, such as electrospinning. Handspun nanofibers retain a larger amount of CNTs than electrospun nanofibers, and the CNTs are easily aligned uniaxially. We attributed these improvements provided by the HS process to simple mechanical stretching force, which allows for orienting the nanofillers along with the force direction without agglomeration, leading to increased contact area between the CNTs and the polymer matrix, thereby providing enhanced interactions. HS is a simple and straightforward method as it does not require an electric field, and, hence, any kinds of polymers and solvents can be applicable. Furthermore, it is feasible to retain a large amount of various nanofillers in the fibers to enhance their physical and chemical properties. Therefore, HS provides an effective pathway to create new types of reinforced nanofibers with outstanding properties.

The electrospinning (ES) technique is an effective and attractive method that has been investigated extensively during the last few decades to fabricate nanofibers from a polymer solution^1^,^2^,^3^. ES produces a variety of polymer fibers or fiber sheets with diameters ranging from a few nanometers to several micrometers and that have a high surface area-to-volume ratio^4^,^5^,^6^. The polymer solution is ejected from the tip of an ES apparatus and forms a fiber structure due to static electrical forces during in-flight solvent evaporation, which leads to the nanofiber structure^7^,^8^. When a sufficiently high voltage is applied to the tip, the highly charged droplets that are ejected from the tip have an electrostatic repulsion force that counteracts the effect of surface tension, allowing the droplets to be stretched out to form nanofibers. The critical point when a stream of liquid erupts from the surface is known as Taylor cone^9^. If the molecular cohesion of the liquid is sufficiently high or if the electrical force is not high enough to overcome molecular cohesion, the Taylor cone cannot be formed. Therefore, the electrical properties of the polymer solution are essential for the successful formation of nanofibers by ES^10^. Thus, the use ES has been highly limited for making nanofibers comprised of polymers that have poor solubility in solvents and low electrical conductivity, such as polyolefins (e.g., polyethylene and polypropylene). In addition, the resulting nanofibers would not have the desired mechanical strength because their diameters typically are small, and the orientation of the polymer chains in the fibers cannot be controlled and optimized.

Recently, we reported a simple and innovative method for fabricating nanofibers, called as handspinning (HS). This method shows promise for overcoming the challenges in ES^11^. HS method was inspired by the process of making cheese or noodles by hand-pulling, or making long and thin fiber-like structure from highly viscous liquid glue using two fingers, grabbing and subsequently pulling out the viscous material with thumb and index finger. Thus, in the HS method, fibers are fabricated completely different mechanism used in ES. In the HS process, a stretching force is used instead of an electrical force, which results in well-oriented polymer fibers along a single axis, whereas ES process typically produces fibers with random orientations. Also, the process relies on simple mechanical force, making high electrical voltage unnecessary, avoiding high cost and excessive use of energy in production. Figure 1e shows that the HS process even worked with the simplest system we devised, i.e., using only two fingers to make nanofibers. More importantly, HS offers a number of options for polymers and solvents because, unlike in the ES process, their electrical properties are not relevant at all. In our previous report^11^, we found that the diameter and surface morphologies of handspun fiber depend on solvent systems and processing conditions to control simple mechanical force. Also, a significant difference in polymer chain conformation between the handspun and electrospun nanofibers was observed, and it was attributed to mechanical stretching force. All related observations strongly indicated that HS provides a versatile and straightforward route to obtain well-defined nanofibers.

Carbon nanotubes (CNTs) have attracted significant scientific and technological attention due to their exceptional mechanical^12^, electrical^13^, and thermal properties^14^. Specifically, CNTs are promising candidates for next-generation reinforcement composite materials, which require extraordinarily high strength with low weight and appropriate electrical properties^15^. According to theoretical predictions, CNTs exhibit 10 to 100 times higher strengths than steel at a fraction of the weight. Specifically, single-walled CNTs show very high Young’s moduli values ranging from 0.32 to 1.47 TPa and tensile strengths ranging from 10 to 52 GPa with a toughness of ~770 J/g^16^. Due to these inherent characteristics, incorporating CNTs into polymers to fabricate nanocomposites has resulted in tremendous improvements in the physical properties of the nanocomposites, e.g., increased tensile strength, increased glass transition temperature, and enhanced fatigue resistance^17^,^18^,^19^. For example, Ko *et al.*^20^. reported that they fabricated continuous CNT-filled nanofiber yarns via ES. They found that the modulus was improved by about 120% when the nanofibers contained 4 wt% of single-wall CNTs. Multi-walled carbon nanotubes (MWCNTs) have been studied extensively because their chemical and physical properties are comparable to those of SWCNTs. In addition, they can be synthesized easily at a large scale. The composites, which are highly-conductive and mechanically-robust materials, possibly used in various applications, e.g., flexible displays and electronic paper^20^.

There have been three significant challenges in the fabrication of CNT-reinforced polymer composites, i.e., (i) the difficulty of concentrating the CNTs in a matrix, (ii) the agglomeration of the CNTs, and (iii) controlling the orientation of the CNTs. Incorporation of a high concentration of CNTs in a polymer matrix without aggregation and localization of the CNTs is difficult because they tend to make a bundle, resulting in their being poor dispersed in the polymer matrix and the deterioration of the properties of the materials^21^,^22^. Thus, retaining high concentrations of both the polymer and CNTs in solution is highly limited, which is critical to control the structure and morphology of nanofibers. Also, it is difficult to control the orientation of nanofillers in polymeric nanofibers with conventional fabrication methods; in the field of composite materials, controlling the orientation of CNTs in polymer matrices has been one of several significant technical challenges^23^,^24^,^25^,^26^. Herein, we demonstrate the use of HS method to address all of these challenges. In the case of ES, the viscoelastic properties are important because the electrical force should overcome the surface tension of the solution^27^. In other words, increasing the concentration of CNTs is limited because it simultaneously increases the viscosity of the solution. However, in HS, an electric field and the related forces are not involved because the process relies only on a simple, mechanical pulling motion (Figs 1 and S1), hence the effect of the concentration of CNTs on the process likely is negligible. In addition, the simple mechanical stretching force in HS is more effective than the complicated, but weak, force induced by the electric field in ES to align the CNTs in the nanofiber. Given this rationale, for the proof of concept, poly(vinyl acetate) (PVAc) was chosen as the polymer matrix to test the potential of HS. Generally, PVAc is used for impact modifiers and processing aids^28^, and PVAc-based composite materials are used extensively to manufacture resin emulsifiers, adhesives, paper, paint, and textile products because of the ease with which it can be used to make a film and its odorless and nonflammable characteristics^29^,^30^. The resulting composite fibers fabricated by the HS process contained highly-concentrated CNTs aligned parallel to the nanofibers. Also, the fibers showed more improved mechanical and thermal properties then electrospun fibers, confirming the positive impacts of the HS method.

## Results and Discussion

### Studies of the morphologies and orientations of MWCNTs in electrospun and handspun nanofibers

**Figure 2:**
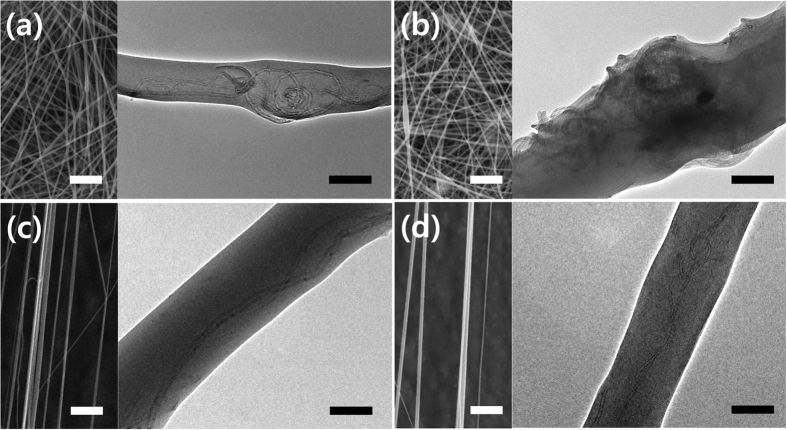
SEM (left) and TEM (right) images of electrospun nanofibers: (**a**) 0.5 wt%; (**b**) 1 wt% of CNTs; handspun nanofibers: (**c**) 0.5 wt%, (**d**) 1 wt% of CNTs. (SEM scale bar = 5 μm, TEM Scale bar = 100 nm).

Figures 2a,b, and S2 show typical SEM images of electrospun PVAc/CNT nanofibers with various concentrations of CNTs. Figures S2f and S3 show the average diameter of the nanofibers and their distributions as a function of the CNT concentration extracted from the SEM images. The diameter of the nanofibers decreased when the concentration of CNTs increased up to 0.5–1.0 wt%. This occurred because CNTs exhibit high conductivity, which results in the reduction of the electrostatic potential, typically making smaller-diameter fibers in the ES process. However, as the concentration of CNTs increases from 2.0 to 5.0 wt%, agglomeration of the superfluous CNTs occurs and, hence, the average diameter becomes larger, as confirmed in the SEM images. The results show that a low concentration of CNTs in the PVAc matrix is critically important in CNT/polymer composite nanofibers because the CNTs cannot be well-dispersed if the concentration is too high^31^.

Figures 2c,d, and S4a–g display typical SEM images of nanofibers fabricated by HS. Contrary to ES, HS has not been investigated thoroughly because it is a completely new method for fabricating composite nanofibers. Initially, handspun nanofibers are aligned quite well compared to electrospun nanofibers, which typically are distributed randomly. Since the force is applied uniaxially to the polymer solution by a pull-out operation, nanofibers are fabricated along the axis in the direction of the operation, leading to well-aligned nanofibers. Figure S4h shows the average diameter and distributions of nanofibers, and it is apparent that a linear increase in the diameter of the nanofibers occurs when the concentration of CNT increases. This is attributed to the corresponding increase in the viscosity of the solution as the amount of CNTs increases in the polymer’s matrix, indicating that viscosity also is a significant factor in determining the diameter of the nanofibers, as is the case for the ES process. This trend was in good agreement with the typical relationship between viscosity and the fibers’ diameter presented in many previous reports^32^. The nanofibers that have a CNT concentration in the range of 0–2 wt% have very-smooth, uniform surfaces. However, at concentrations greater than 5 wt%, the edges of nanofibers were somewhat rougher than the edges of the nanofibers with lower concentrations, as indicated by the red circles in Figures S4e, 4f, and 4g, but the difference was not significant. The roughness likely was due to some aggregation of the CNTs in PVAc matrix, but we noted that the handspun nanofibers did not show any bead-on-string structure along the fiber as electrospun nanofibers do; the beads along the fiber commonly are observed in electrospun nanofibers, even at low concentrations of CNT. Therefore, the results strongly suggested that, using the HS method, CNTs can be highly concentrated in the polymer matrix with well-aligned, low edge-roughness fibers, which could not be achieved using the ES method.

The microstructure of the nanofibers was investigated further using TEM. Figure 2 shows typical TEM micrographs (right) corresponding to SEM micrographs (left) for 0.5 and 1 wt% CNT/PVAc nanofibers prepared by both ES and HS. (TEM images of nanofibers with higher concentrations of CNTs are presented in Figures S6 and S7). The CNTs inside the fibers were observed in TEM images for both methods. The TEM images clearly indicate that the orientation of the CNTs in the PVAc matrix was dependent on the fabrication method. In handspun nanofibers, the CNTs were aligned along the PVAc fibers (Fig. 2c and d), but for electrospun nanofibers, the CNTs were entangled, bent, and incompletely wrapped by the PVAc matrix (Fig. 2a and b). The results indicated that the force applied during the pull-out process affected the distribution of the filling materials in nanofibers. In the ES process, a high voltage typically is used to create an electrically-charged jet of polymer solution, inducing charges on the surface of polymer solution. Mutual repulsion and the contraction of the surface charges to the counter electrode cause a force directly opposite that of the surface tension^32^. When the repulsive electrostatic force exceeds the surface tension, a charged jet of the fluid is ejected from the tip, which undergoes an elongation process due to its instability, resulting in a long, thin structure of the fibers from the polymer solution. In this process, the CNTs are not expected to be aligned because the electrical treatment cannot be translated to mechanical force to stretch the CNTs. The HS process, however, relies solely on a mechanical force applied to the polymer solution. Simply pulling two plates where the polymer solution present leads to well-defined nanofibers with a parallel orientation and uniaxial alignment of the CNTs to the fibers, all of which are induced by simple mechanical effects.

### Enhanced mechanical properties of the nanofibers

**Figure 3:**
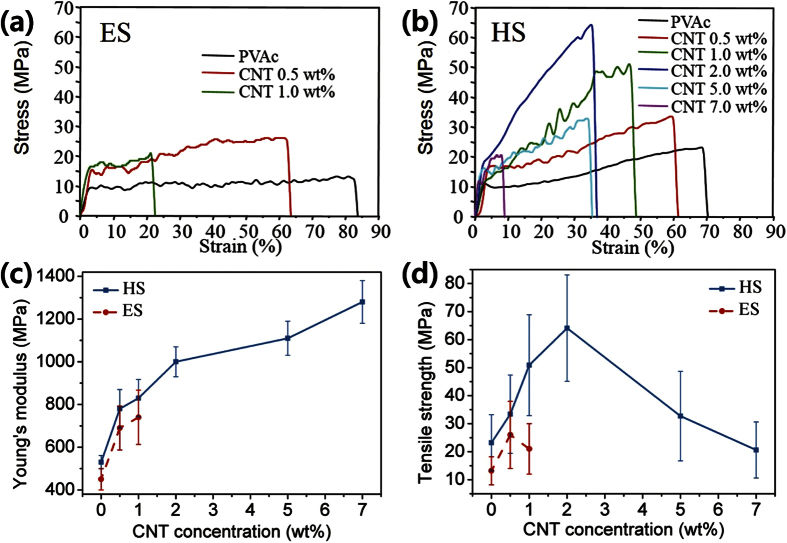
Stress-strain curves measured with the various CNT concentrations (**a**) single electrospun nanofibers; (**b**) single handspun nanofibers; (**c** and **d**) tensile strength and Young’s modulus of single electrospun and handspun nanofibers extracted from the stress-strain curves.

To investigate the effect of the fabrication method on the nanofibers’ mechanical properties, Young’s modulus and the tensile strength of a single nanofiber were measured from strain-stress curves of the electrospun and handspun samples as the concentration of CNTs was varied (Fig. 3 and Table 1). The electrospun PVAc nanofibers without fillers had a tensile strength and a Young’s modulus of 13.2 ± 5.0 and 450 ± 49 MPa, respectively, while those of the handspun PVAc nanofibers were 23.2 ± 10.1 and 530 ± 31 MPa, respectively, corresponding to ~180 and 120% increases, respectively, when the fabrication method was changed from ES to HS. This indicated that the mechanical stretching force in the HS process induced the alignment of the polymer chains. The important aspects that affect the strength of nanofibers are the alignment of polymer chains along the axis of the fibers and the degree of crystallinity. In our previous report^11^, it was found that the HS process produced more stretched fibers than the ES process. However, the degree of crystallinity was not affected critically by the spinning method, *i.e.,* electrospinning or handspinning, due to their relatively low crystallinity. Thus, the HS process is beneficial in that it enhances the tensile strength of the fibers by inducing polymer chain alignment, which the ES process cannot do. Also, the incorporation of CNTs should have a significant effect due to the resulting enhancement of the mechanical properties of the nanofibers. Increasing the amount of CNTs in the PVAc matrix dramatically increased Young’s modulus and tensile strength (Fig. 3c and d). Note that we were unable to use our apparatus to characterize electrospun nanofibers with CNT concentrations greater than 2 wt% or handspun nanofibers with CNT concentrations greater than 10 wt% due to their poor mechanical properties. Using the ES method, 0.5 wt% loaded PVAc/CNT nanofiber had higher tensile strength and modulus, i.e., 26.3 ± 12.1 MPa and 690 ± 103 MPa, respectively, than the PVAc nanofiber. However, when the concentration was increased to 1 wt%, the tensile strength decreased to 21.5 ± 9.2 MPa, while the modulus increased to 740 ± 127 MPa. Because CNT aggregates were formed at concentrations greater than 1 wt%, as confirmed in the electron microscopic studies (Figures S2 and S4), the tensile force was not distributed evenly along the fiber; rather, it was focused on local points where the CNT aggregates were located in the nanofiber, leading to lower tensile strength. However, the handspun nanofibers did not exhibit the degradation of tensile strength at CNT concentrations less than 2 wt%. The tensile strength and the modulus were 33.4 ± 13.9 MPa and 780 ± 90 MPa, respectively, when the concentration of CNTs was 0.5 wt%, and increasing the concentration of CNTs from 1 wt% to 2 wt% resulted in an increase in the tensile strength from 50.9 ± 18.3 to 64.1 ± 19.1 MPa and an increase in the modulus from 830 ± 87 to 1000 ± 71 MPa. Young’s modulus of the samples with CNT concentrations of 5 and 7 wt% increased to 1110 ± 83 and 1280 ± 102 MPa, respectively, indicating the nanofibers became more rigid even though the tensile strengths decreased to 32.7 ± 16.2 and 20.6 ± 10.4 MPa, respectively. When the CNT concentration was increased from 5 to 7 wt%, the breaking point of a single nanofiber changed 70 to <10%. This indicated that the nanofibers cannot withstand the application of an elongation force, which means the tensile strength was inversely proportional to the concentration of the filler^33^. In comparison to the conventional ES method, the HS method enhanced the mechanical properties of the nanofibers by a simple change in the fabrication method. The ES method could not be used to make highly-concentrated CNTs in PVAc fiber, but the HS method, in which the concentration of CNT could be increased, achieved a Young’s modulus that was 1.8 times was greater than that of the ES method. At the same CNT concentration for both methods, the tensile strength 2.4 times greater for the HS method, making it a powerful tool to attain strong nanofibers when the orientation of the CNTs is important.

### Differences in crystallinity and thermal properties in nanofibers

**Figure 4:**
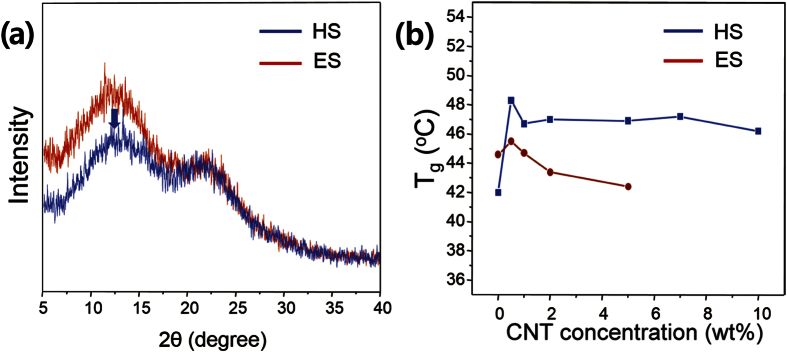
(**a**) Representative XRD spectra; (**b**) plots of T_g_ as a function of CNT concentration of handspun and electrospun nanofibers.

We also conducted studies to compare HS and ES in order to understand how the fabrication method affects the structures and properties of the nanofibers that are produced. Figures 4a and S8 show the wide-angle X-ray diffraction spectra of handspun and electrospun PVAc/CNTs nanofibers. Broad peaks were observed at 2θ = 13° from both the handspun and electrospun samples, typically indicating semi-crystalline regions attributable to the ordering of the polymer chains by hydrogen bonding^34^,^35^. Although both ES and HS fibers had relatively low crystallinity due to the rapid fabrication process, it was noted that the handspun fiber exhibited lower intensity than the electrospun fiber, and this was attributed to the decrease of the overall crystallinity of the PVAc/CNT nanofibers^36^; in other words, the handspun sample had a greater amorphous portion. There could be two scenarios to explain the phenomena. First, it is known that molecular ordering occurs due to the electrostatic stretching force induced in ES process^37^,^38^. This implies that the higher stretching force in HS makes the polymer chains more ordered, resulting in breaking the semi-crystalline structure. Second, hydrogen bonding between the ester groups, which were formed in the polymer chains and in the carboxylic acid groups on the CNTs by pre-treatment with acid, could disrupt the crystallinity of the PVAc^39^,^40^. Thus, these results emphasize the significance of understanding the interaction in the composite.

Differential scanning calorimeter (DSC) was performed to determine the glass transition temperature (T_g_), which could provide better understanding the interaction by the hydrogen bonding of the carbonyl group in the PVAc chains to CNTs. Figure S9 shows the DSC curves of the CNT/PVAc composite nanofibers with various concentrations of CNT, exhibiting a single T_g_ over the entire CNT concentration range. As shown in Fig. 4b, the addition of CNT to PVAc at the concentrations up to 0.5 wt% resulted in an increase in T_g_ of about 6 °C, which was attributed to the addition of inorganic filler to the polymer^41^,^42^. For example, in the PVAc /TiO_2_ composite membrane, the increase of T_g_ that occurred when TiO_2_ was added occurred due to hydrogen bonding between the hydroxyl groups on surface of the TiO_2_ and the sulfone or ether groups of PVAc^43^. The increase of T_g_ also indicated that the CNTs were dispersed well in the PVAc fiber^41^. However, the electrospun nanofibers showed a decrease in T_g_ as concentration of CNTs increased. At concentrations greater than 0.5 wt%, the CNTs aggregated, as observed in the morphological studies, leading to a reduction in the value of T_g_ similar to the trend for the mechanical properties. However, the decrease in the value of T_g_ was not observed in the handspun nanofibers; the addition of 0.5 wt% CNTs led to an increase in T_g_ from 42 to 48 °C, and then, in spite of further addition of CNTs up to 10 wt%, T_g_ became saturated at ~47 °C. These results strongly suggested that (i) the CNTs interacted strongly with PVAc and (ii) the CNTs were well-dispersed in the PVAc nanofiber, in good agreement with electron microscope study.

Controlling the orientation of the well-dispersed CNTs in nanofibers has been of interest in electrospinning, e.g., ES of a CNT/polymer solution in a magnetic field. However, HS is a much simpler technique, and it reduces possible complexities in the spinning process while effectively achieving comparable dispersion of CNTs in the matrix with a controlled orientation. Therefore, HS should be a useful method to improve a range of properties in nanofibers, providing a solution to overcome certain limitations of ES, such as the poor mechanical properties due to the limited amount of CNTs. Furthermore, HS provides a controllable way to prepare composite nanofibers with highly concentrated and well-aligned nanofillers while simultaneously offering several options for polymers and solvents, since their electrical properties are not relevant at all. We note that the throughput of handspinning could be low compared to the conventional spinning method; however, the results provided in this study are the proof of concept to fabricate well-defined composite nanofibers. It should be possible to improve the instrumental set-up, achieve high efficiency, and produce high quality nanofibers simultaneously. This paper is the first to report the use of HS to incorporate CNTs into nanofibers so that they are aligned along the axes of the nanofibers. This new fabrication method provides a versatile, straightforward, facile, and effective pathway to create well-defined, CNT-reinforced polymeric nanofibers that have outstanding properties.

## Conclusions

The innovative method, *Handspinning,* inspired by other processes in our daily lives, offers an effective and facile route for fabricating highly-concentrated and well-orientated CNT/PVAc nanofibers. The simple ‘pulling-out’ motion generates mechanical stretching force in the polymer solution, resulting in long, thin nanofiber structures. Handspun nanofibers exhibited significantly-improved thermal and mechanical properties when compared with ES nanofibers. The HS nanofibers had evenly-distributed CNTs with minimal agglomeration as well as uniaxial alignment of the CNTs with the nanofibers, which led to more significant interaction between the CNTs and the polymer than was the case for the ES nanofibers. HS nanofibers had values of Young’s modulus that were about 1.8 times greater than Young’s modulus for ES nanofibers. This was accomplished by increasing the concentrations of the CNTs, and, at the same CNT concentration for both methods, the tensile strength for the HS nanofibers was 2.4 times better than that for the ES nanofibers. Also, the HS nanofibers had a higher T_g_ than the ES nanofibers, confirming that the aggregation of CNTs was not substantial in the HS method. Therefore, it is clear that the stretching force applied during the HS process produces a uniaxial orientation of individual CNTs along with stretching direction without significant aggregation, resulting in an increment of surface area where the CNTs and the polymer matrix made contact. Mimicking a commonly-observed phenomenon, i.e., making fiber-like structures by pulling two fingers out of a viscous material, was beneficial in improving various properties of nanofibers. It provided a solution that allowed us to overcome the limitations and issues in ES, such as the low mechanical properties due to the limited amount of nanofillers in the polymer matrix. Since HS is a simple, straightforward, and versatile method that is universally applicable to any kinds of polymers and solvents, it enables us to create a new type of reinforced polymeric composite nanofibers with exceptional properties.

## Experimental

### Preparation of Polymer/CNT solutions

To improve the miscibility of CNTs with PVAc, pristine MWCNTs (Baytube C150P, Bayer Material Science AG, Germany) were added and sonicated in a mixture of sulfuric acid and nitric acid (3:1 H_2_SO_4_:HNO_3_) for 3 hr at ~50 °C^44^ and stored for one day. The acid-treated MWCNTs were washed four or five times with deionized water until the pH became neutral, followed by drying under vacuum. The resulting MWCNTs were redispersed in dimethylformamide (DMF) (Wako Pure Chemical Industries, Ltd. >99.5%) with various concentrations by sonication for 3 hr and used as a stock solution. PVAc (Mw ~ 500 kg/mol, Sigma Aldrich, Co., USA) was dissolved in a certain amount of DMF, and, then, the desired volume of the MWCNT dispersion was added to control the concentration of MWCNTs from 0 to 10 wt% relative to the amount of PVAc in solution, where the concentration of PVAc was 18 wt%. The mixture was homogenized further by sonication for 30 min.

### Nanofiber fabrications

For ES, a high-voltage power supply (Har-100*12, Matsusada Co., Tokyo, Japan), capable of generating voltages up to 80 kV, was used as the source of the electric field. The PVAc/CNT solutions were supplied through a plastic syringe to a capillary tip with an inner diameter of 0.6 mm. A copper wire connected to a positive electrode (anode) was inserted into the PVAc/CNT solution, and a negative electrode (cathode) was connected to a metallic collector. The voltage was fixed at 10 kV, and the distance between the capillary tip and the metal collector was 15 cm. The resulting samples were dried under vacuum overnight. For HS, we built the apparatus shown in Figure S1^11^. The apparatus was designed to control various processing parameters, i.e., pulling away speed (PAS, cm/s), pulling away distance (PAD, cm), and plate area (PA, cm^2^). In this work, PAS, PAD, and PA were controlled accurately to be 40 cm/s, 13 cm, and 66 cm^2^, respectively. The typical volume of the solution was 10 mL. The resulting samples were dried under vacuum overnight. To optimize the processing conditions, we conducted the handspinning process by varying PAS from 30 to 50 kV, and the PAD was varied from 10 to 18 cm. All of the samples of the composite nanofibers were prepared under optimum conditions. All samples of the nanofibers were prepared with the same process parameters. At the conditions specified above, confirmed the repeatability of the handspinning process to fabricate uniform nanofibers with multiple spinning operations.

### Characterization

The morphologies of both HS and ES PVAc/CNTs nanofibers were observed with scanning electron microscopy (SEM, JSM-6010LA, JEOL, Japan). The average diameter and distribution of the nanofibers were measured from the SEM micrographs using image analysis software (Image J, version 1.49). To obtain the distribution of diameters and their average value, at least 50 points in each SEM image of the corresponding sample were selected randomly and evaluated in the diameter range of 100–1100 nm. Transmission electron microscopy (TEM) (JEM-2100 JEOL Japan, accelerating voltage 120 kV) was utilized to examine the CNTs inside the resulting nanofibers. The mechanical properties of single nanofibers were characterized using a specially-developed tensile test machine (FITRON NFR-1000, RHESCA Co., Japan, maximum loading capacity: 500 mN, maximum stroke: 20 mm, loading speed: 5–20 μm/s, displacement sensitivity: 1.0 μm, loading sensitivity: 1.0 μN). The glass transition temperatures of the nanofibers were measured with a differential scanning calorimeter (DSC, Thermo plus DSC8230, Rigaku Co., Ltd., Japan) in argon gas with a heating rate of 5 °C/min from 20 to 80 °C. The wide-angle X-ray diffraction (XRD) experiments were performed at room temperature using a Rotaflex RTP300 X-ray diffractometer (Rigaku Co., Japan) operating at 50 kV and 200 mA. Nickel-filtered Cu Kα radiation was used as the X-ray source, and diffraction was detected over an angular range of 5 to 40°.

## Additional Information

**How to cite this article**: Lee, H. *et al.* Handspinning Enabled Highly Concentrated Carbon Nanotubes with Controlled Orientation in Nanofibers. *Sci. Rep.* **6**, 37590; doi: 10.1038/srep37590 (2016).

**Publisher’s note:** Springer Nature remains neutral with regard to jurisdictional claims in published maps and institutional affiliations.

## Supplementary Material

Supplementary Information

## Figures and Tables

**Figure 1:**
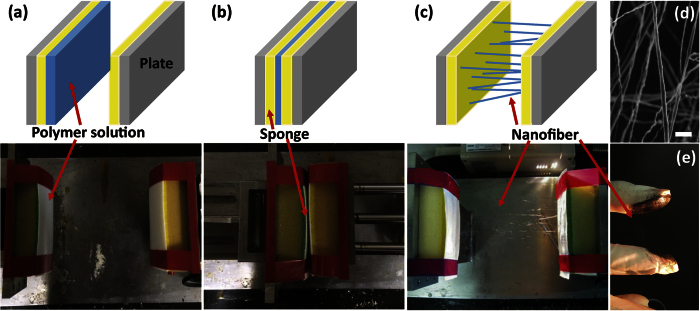
(**a–c**) Schematic illustrations and corresponding photographs of the nanofiber fabrication process via handspinning; (**d**) representative SEM image of a handspun nanofiber (scale bar = 10 μm); (**e**) photograph showing hand-made, CNT-reinforced nanofibers using two fingers.

**Table 1:** Young’s modulus and tensile strength of single PVAc/CNTs nanofibers fabricated by ES and HS with various weight percentages of CNTs.

		CNTs (wt%)					
		0	0.5	1.0	2.0	5.0	7.0
ES	Young’s modulus (MPa)	450 ± 49	690 ± 103	740 ± 127	—	—	—
	Tensile strength (MPa)	13.2 ± 5.0	26.3 ± 12.1	21.5 ± 9.2	—	—	—
HS	Young’s modulus (MPa)	530 ± 31	780 ± 90	830 ± 87	1000 ± 71	1110 ± 83	1280 ± 102
	Tensile strength (MPa)	23.2 ± 10.1	33.4 ± 13.9	50.9 ± 18.3	64.1 ± 19.1	32.7 ± 16.2	20.6 ± 10.4
