# Supplementary material for: Handspinning Enabled Highly Concentrated Carbon Nanotubes with Controlled Orientation in Nanofibers
Source: Sci Rep. 2016 Nov 23;6:37590. doi: 10.1038/srep37590 (PMC5120309; doi:10.1038/srep37590)
Supplement: Supplementary Information [file srep37590-s1.pdf]

Supporting Information

Handspinning Enabled Highly Concentrated Carbon Nanotubes with Controlled Orientation in Nanofibers

Hoik Lee,[†,*] Kei Watanabe,[†] Myungwoong Kim,[‡] Mayakrishnan Gopiraman,[†] Kyung-Hun Song[||], Jung Soon Lee[§] and Ick Soo Kim[†],*

[†]*Nano Fusion Technology Research Lab, Division of Frontier Fibers, Institute for Fiber Engineering (IFES), Interdisciplinary Cluster for Cutting Edge Research (ICCER), Shinshu University 3-15-1, Tokida, Ueda, Nagono 386-8567, Japan*

[‡]*Department of Chemistry, Inha University, Incheon 22212, Korea*

[||]*Department of Clothing and Textiles, Pai Chai University, Daejeon 35345, Korea*

[§]*Department of Clothing and Textiles, Chungnam National University, Daejeon 34134, Korea*

**Corresponding authors: Hoik Lee (lee-hoik@shinshu-u.ac.jp) and Ick Soo Kim (kim@shinshu-u.ac.jp)*

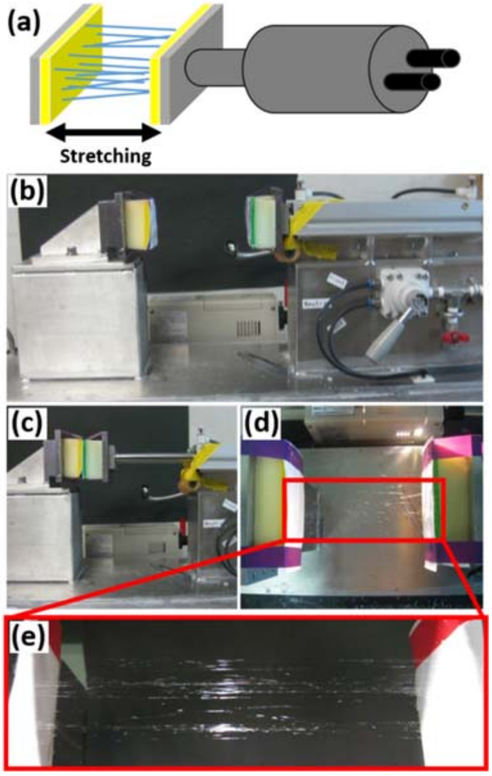

Figure S1. (a) Schematic drawing and (b) photograph of home-built handspinning apparatus, (c) polymer solution is applied between two plates, (d) nanofibers are fabricated by pulling two plates out, and (e) zoomed photograph showing nanofibers.

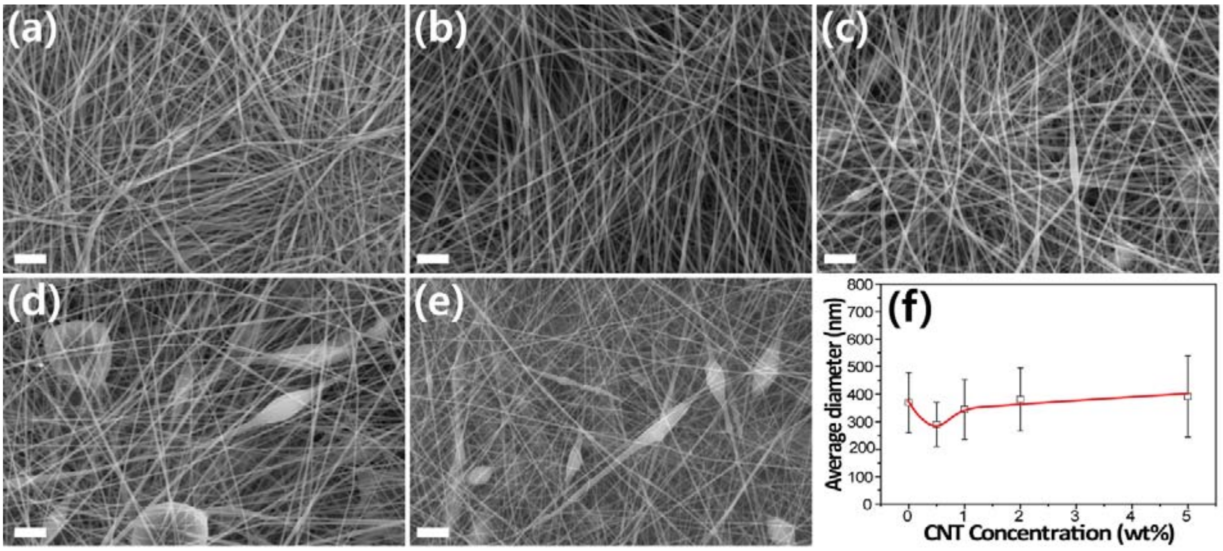

Figure S2. SEM images of electrospun nanofibers as a function of CNT concentration, (a) only PVAc, (b) 0.5 wt%, (c) 1 wt%, (d) 2 wt%, (e) 5 wt% of CNT concentration, and (f) the plot of average diameter as a function of CNT concentration. Red line is the guide to the eye. (Scale bar = 5 μm)

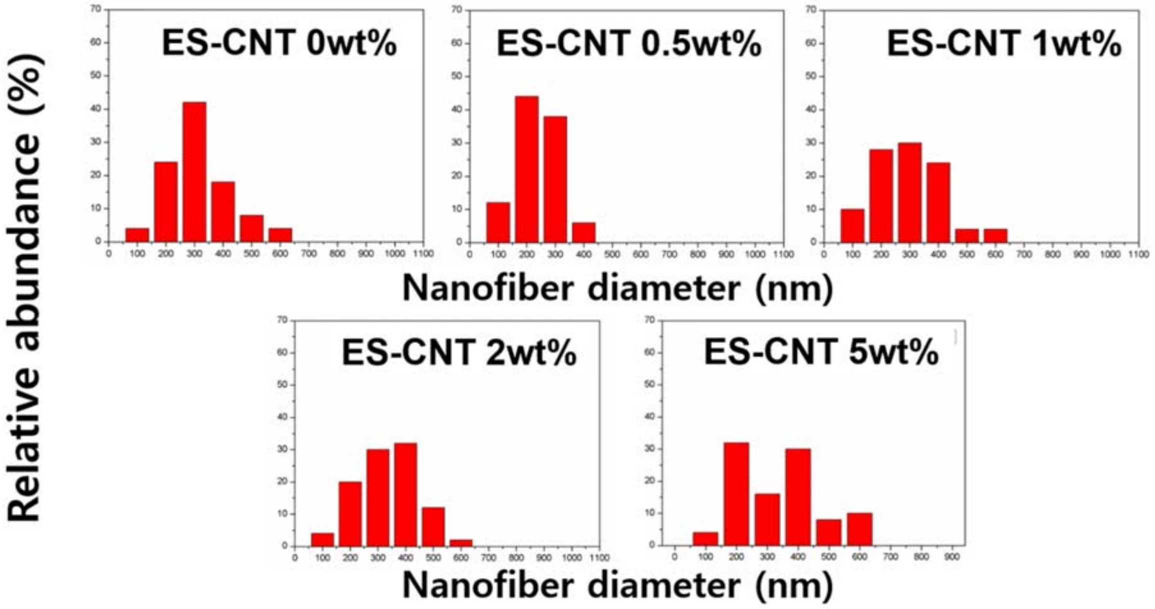

Figure S3. Diameter distributions of electrospun nanofibers as a function of CNT concentration.

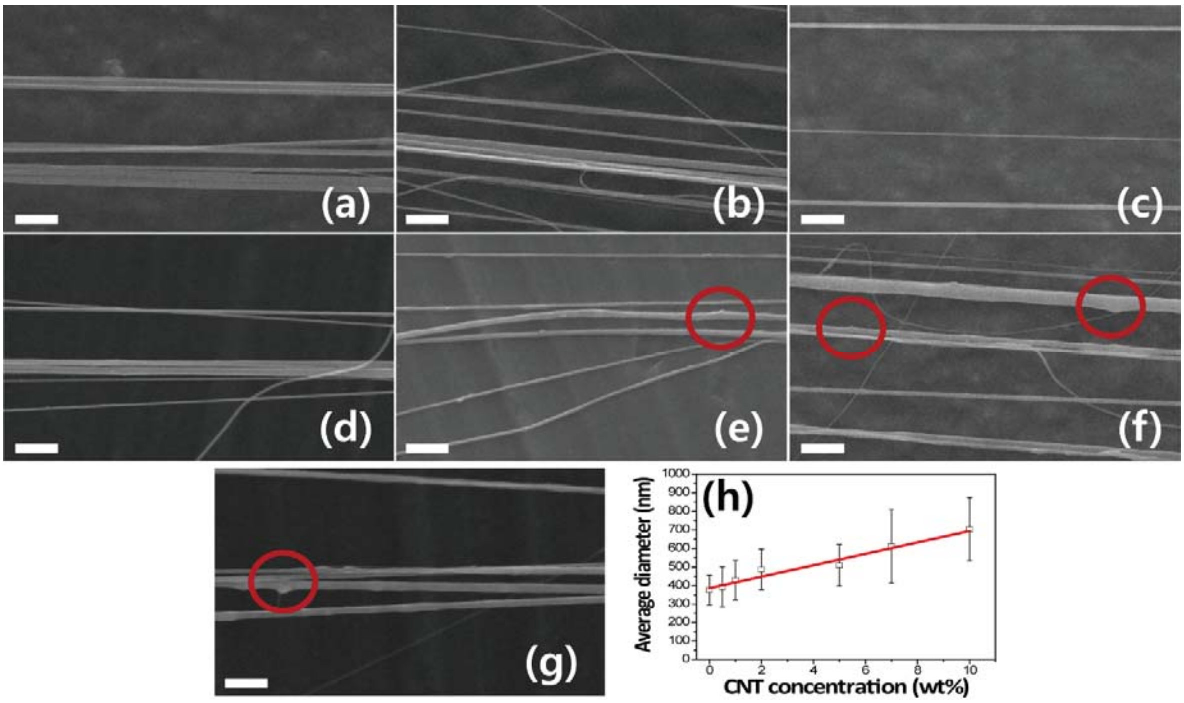

Figure S4. SEM images of handspun nanofibers as a function of CNT concentration, (a) only PVAc, (b) 0.5 wt%, (c) 1 wt%, (d) 2 wt%, (e) 5 wt%, (f) 7 wt%, (g) 10 wt% of CNT concentration, and (h) the plot of average diameter as a function of CNT concentration. Red line is the guide to the eye. (Scale bar = 5 μm)

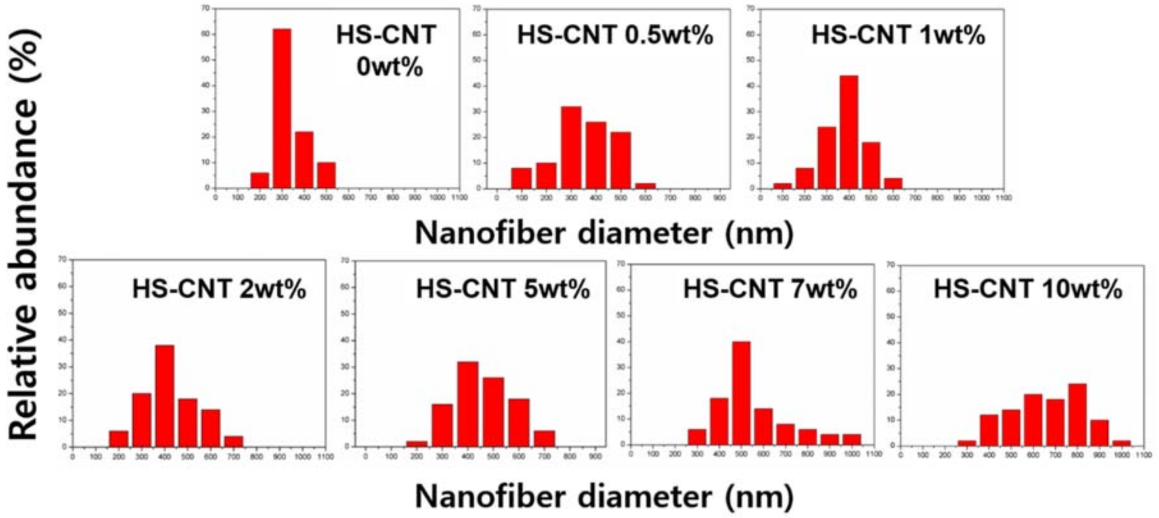

Figure S5. Diameter distributions of handspun nanofibers as a function of CNT concentration.

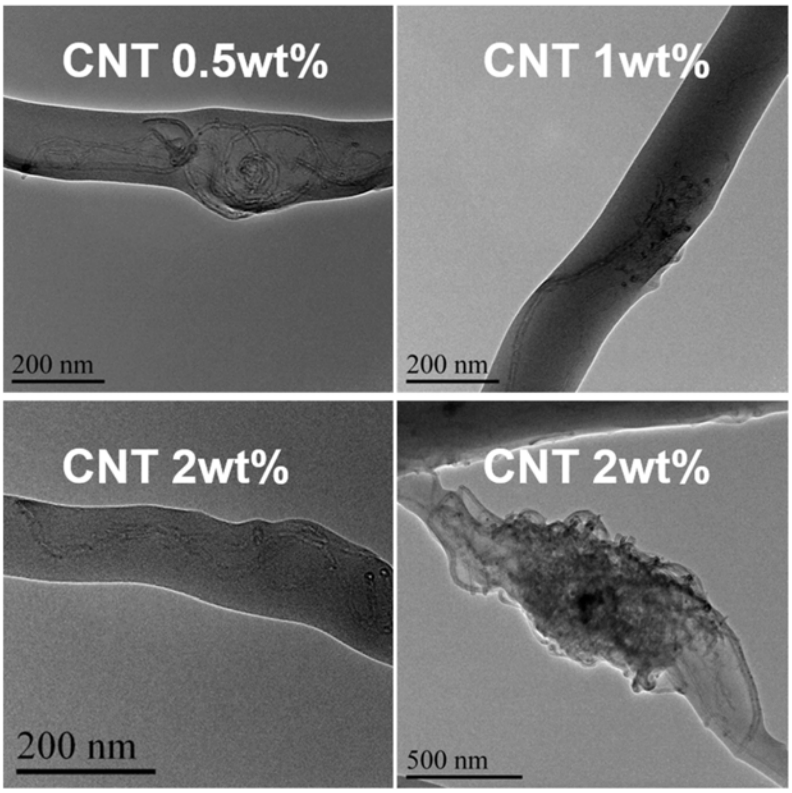

Figure S6. TEM images of PVAc/CNTs nanofibers fabricated by ES.

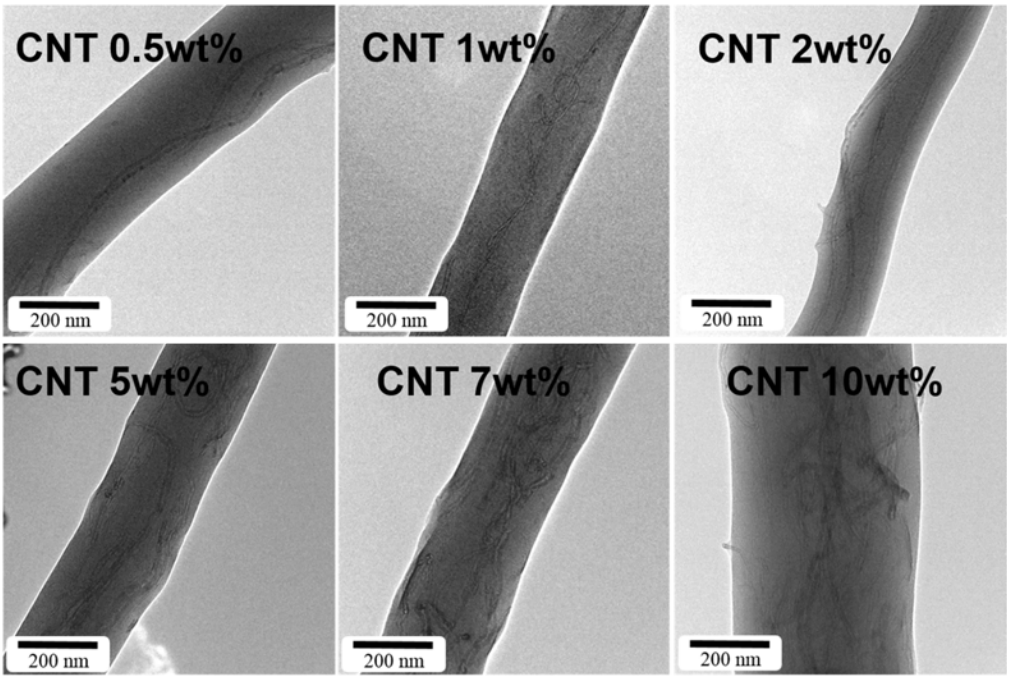

Figure S7. TEM images of PVAc/CNTs nanofibers fabricated by HS.

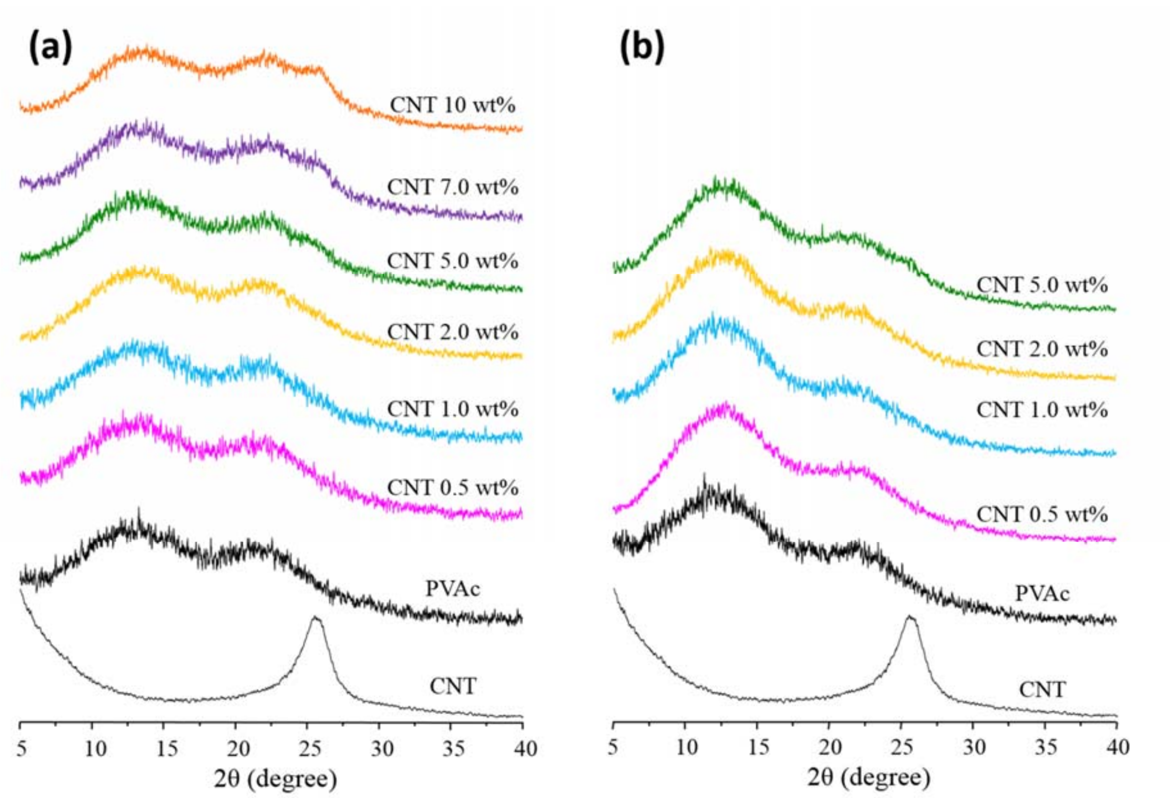

Figure S8. Wide angle X-ray diffraction spectra of (a) handspun and (b) electrospun PVAc/CNTs nanofibers.

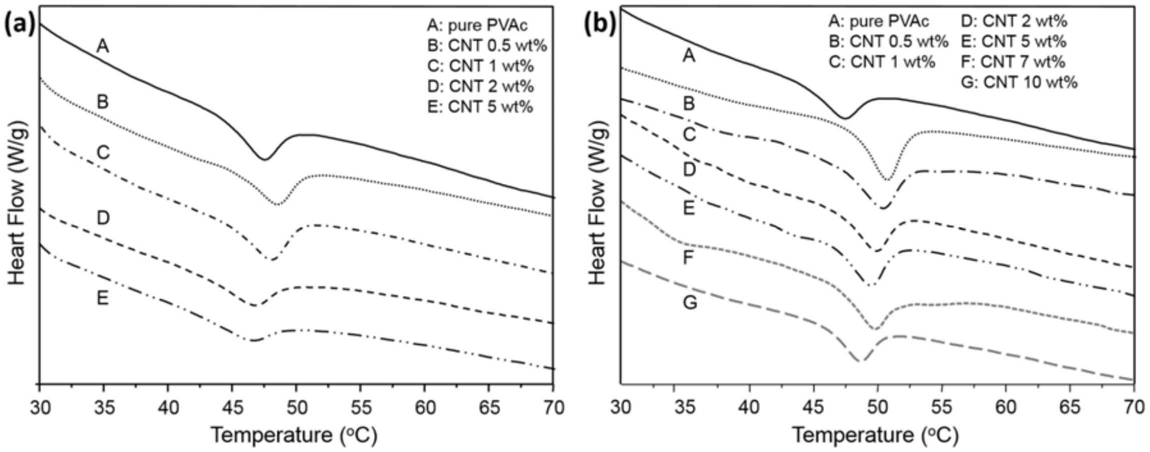

Figure S9. DSC curves of CNT/PVAc composite (a) electrospun and (b) handspun nanofibers with the variation of CNT concentration.
